# Editorial: Plant bioactive compounds as modulators of oncogenic signalling pathways in cervical cancer

**DOI:** 10.3389/fphar.2026.1788689

**Published:** 2026-02-05

**Authors:** Pratibha Pandey, Arif Hussain, Fahad Khan

**Affiliations:** 1 University Centre for Research and Development, Chandigarh University, Mohali, Punjab, India; 2 School of Life Sciences, Manipal Academy of Higher Education, Dubai, United Arab Emirates; 3 Department of Community Medicine, Saveetha Medical College and Hospital, Saveetha Institute of Medical and Technical Sciences, Chennai, Tamil Nadu, India

**Keywords:** anticancer, cancer therapy, cell signaling pathway, cervical cancer, plant derived compounds

## Introduction

Cervical cancer is a highly aggressive types of cancer and leading cause of death in women (Sung et al., 2021). The disease progression from primary to advance metastatic stage is mainly govern by persistent infection to human papillomavirus (HPV) (Okunade, 2020). Beside this, a number of possible risk factors of cervical cancer progression include smoking, early sexual activity, genetic factors and other viral infections. There are more than 100 types of HPV have been identified till date as high risk and low risk type. Among these, HPV E6 and E7 are the most pathogenic and mainly responsible for oncogenic phenotype in cervical cancer (Yang et al., 2022). Several oncogenic signaling pathways including PI3K/Akt, JAK/STAT, TGF-β, Notch, MAPK/p38, NF-κB, have been recognized to be involved in cervical cancer pathogenesis by regulating gene expression. A better understanding of these molecular pathways and functions could offer the new possibilities of targeting cervical cancer (Rasi Bonab et al., 2021; Hazazi et al., 2024). The conventional treatment approaches including chemotherapy, surgery, radiotherapy remains ineffective and caused severe adverse reactions in cancer patients. Thus, there is an urgent need for novel therapeutic agents which are more effective and less toxic (Burmeister et al., 2022). In this regard, plant derived compounds have been considered as potent candidate for anticancer drug development by targeting various molecular and cellular pathways (Cakir et al., 2025).

Natural compounds exhibit unmatched structural diversity owing to the metabolic repertoire of plants, animals and microbes. These evolutionarily developed molecules have complicated and unique structures that much more complex than those of synthetic compounds. The unique chemical properties of plant derived natural compounds facilitate them to interact with a diverse range of biological targets in several distinct manners, making them particularly useful for anticancer drug discovery. By controlling these oncogenic signaling pathways, plant compounds such as volatile oils, flavonoids, glycosides, phenols, esters, and alkaloids have been demonstrated to have antitumor properties. The potential action of these plant derived compounds against various types of carcinomas including cervical cancer is noteworthy (Hashem et al., 2022). Plant-based bioactive compounds help in controlling many cellular processes and signaling pathways by regulating epigenetics in cervical cancer and affecting inflammation, cell proliferation, and apoptosis (Khairkhah et al., 2022). Numerous preclinical investigations have demonstrated that using these bioactive substances is both safe and bearable (Hosseini et al., 2025). Therefore, the goal of this Research Topic is to find new natural substances found in plants and their molecular mechanism of action by focusing on abnormal cell signaling pathways that contribute to the development of cervical cancer. The experimental research findings and proof in this Research Topic will assist scientists and researchers in understanding the diverse beneficial impacts of many types of natural products for the creation of novel and safe drugs for potential cancer therapy.

## Bioactive compounds as therapeutic agent in HPV related cancers

Bioactive molecules derived from natural sources are being investigated as adjuvant therapy for HPV related disorders, while drug repurposing and combination therapy present economical avenues to expand treatment alternatives. Muddather et al. showed that the harmine alkaloid produced from Peganum harmala significantly inhibits cell proliferation and metastasis in HPV-positive cervical and head-neck cells. Harmine was investigated for its significant anticancer efficacy and tumor selectivity to elucidate its potential molecular basis in HPV-positive cells. Results demonstrated that harmine treatment resulted in repressed cell proliferation, apoptotic induction and cell cycle arrest in cancer cells by increasing the stability of tubulin polymers. Moreover, harmine and root extract also shown anti-invasive properties by significantly suppressing cell migration.

In a meta-analysis study, Sun et al. demonstrated the clinical efficacy and safety profile of Astragalus polysaccharide (APS) isolated Chinese medicinal herb Astragalus membranaceus in combination with standard therapy against cervical cancer. The authors conducted a meta-analysis of retrieved data and found that APS can work as adjuvant with chemotherapy and radiotherapy in cervical carcinoma. This adjuvant therapy of APS also enhanced the short effectiveness of these conventional therapies. Furthermore, this APS combination therapy is resulted in better physical state, regulated immune function, low levels of tumor markers and minimum side effects of standard cancer therapy. Altogether, this study suggested that APS can be considered as adjuvant therapeutic agent for cervical cancer management.

## Bioactive compounds as oncogenic signaling modulators

A narrative review study by Asiri et al. highlighted the role of three phytochemicals (curcumin, EGCG and apigenin) in disrupting the oncogenic signaling pathways in cervical cancer. They mainly emphasize on preclinical and clinical findings of these natural compounds as well as their combinatorial studies with conventional therapies. These compounds have shown promising anti-cervical cancer effects by modulating PI3K/Akt, JAK/STAT, Wnt/β-catenin and NF-κB pathways in preclinical reports. These pathways are mainly involved in the tumor progression, metastasis and invasion. This study also provided an overview of synergistic combination of these natural compounds with standard therapies to sensitize drug resistant cells and enhance efficacy in cervical cancer. Moreover, this study elaborates on the limitations associated with the bioavailability and clinical translation of natural compounds.

Another review article by Zeng et al. published in this Research Topic explores the anti-colorectal potential of Traditional Chinese Medicine (TCM) by disrupting Wnt/β-catenin pathway. Their review study highlighted the recent reports demonstrating the role of Wnt/β-catenin pathway in the pathogenesis, angiogenesis, cell cycle distribution and metastasis of colorectal cancer (CRC). Authors compiled the 5 years data on the molecular insights of bioactive molecules of TCM in modulating the Wnt/β-catenin pathway in CRC. The study focused on the TCM monomers, formulations and small-molecule inhibitors in association with Wnt/β-catenin pathway mediated CRC treatment. Moreover, challenges in translating these bioactive compounds of TCM from bench to bedside were also discussed.

In conclusion, modulation of cell signaling pathways is a crucial step in the advancement and development of several types of carcinomas including cervical cancer. Specific targeting of these dysregulated pathways or their key targets by natural compounds could lead us to the development of novel anticancer agents. The studies published in this Research Topic suggested the involvement of dysregulated cell signaling pathways in tumor progression, metastasis, and angiogenesis across various carcinomas. This Research Topic also highlighted the potential of plant derived natural compounds in modulating the dysregulated signaling cascades in different types of preclinical cancer models. Additionally, exploration of various plant derived compounds have provided a basis for development of anticancer drugs by targeting oncogenic signaling pathways. However, challenges associated with bioavailability, dose optimization of compounds, and specific targeting of these pathways are still exist. Future research should be focused to overcome these challenges and provide a better targeting approach for cancer management.

## Challenges and future prospects

Plant derived natural compounds are regarded as a principal source for drug development owing to their enormous structural and physiochemical variations. Various natural compounds that prevent carcinogenesis have unique and common traits. The multitargeted approach of natural compounds allow them to efficiently address the molecular intricacies of cancer, suggesting their substantial potential as anticancer drugs. However, anticancer agents derived from natural substances require significant advancements before clinical application. The prospective use of many natural substances in cancer prevention has been thoroughly investigated. Growing *in vitro* and *in vivo* evidence demonstrated the significant efficacy of these compounds in cancer prevention, prompting scientists to initiate clinical trials. Currently, there are few clinical investigations utilizing natural compounds as anticancer agents, primarily focusing on theor combination with other natural compounds or chemotherapeutic medicines. These investigations have emphasized the safety, efficacy and pharmacokinetics of natural compounds. Although natural compounds have low bioavailability, they can be used as platform to generate synthetic drugs with superior pharmacokinetic features. Another direction for advancement is the development of new drug delivery approaches and drug-loading carriers that can precisely regulate drug distribution to target sites and minimize systemic adverse reactions. Additionally, there are still significant gaps in our knowledge regarding the safety profiles, mode of action and their relative benefits over conventional therapies. To improve clinically feasible treatments based on natural compounds, future research should optimize structures, learn how they work, evaluate their safety thoroughly, and compare their effectiveness.
